# Level and correlates of physical activity among children and adolescents with juvenile idiopathic arthritis compared to controls: results from a German nationwide prospective observational cohort study

**DOI:** 10.1186/s12969-024-00976-2

**Published:** 2024-03-20

**Authors:** Florian Milatz, Sandra Hansmann, Jens Klotsche, Martina Niewerth, Tilmann Kallinich, Frank Dressler, Johannes-Peter Haas, Rainer Berendes, Gerd Horneff, Markus Hufnagel, Frank Weller-Heinemann, Daniel Windschall, Ralf Trauzeddel, Moritz Klaas, Hermann Girschick, Prasad T. Oommen, Ivan Foeldvari, Serdar Mustafa Cantez, Annette F. Jansson, Matthias Hartmann, Joachim Peitz-Kornbrust, Kirsten Minden

**Affiliations:** 1 Programme area Epidemiology and Health Services Research, Deutsches Rheuma-Forschungszentrum Berlin, ein Institut der Leibniz-Gemeinschaft, Berlin, Germany; 2 Department of Neuropediatrics, Developmental Neurology and Social Paediatrics, University of Tuebingen, Tuebingen, Germany; 3 Department of Paediatric Respiratory Medicine, Immunology and Critical Care Medicine, Charité – Universitätsmedizin Berlin, corporate member of Freie Universität Berlin and Humboldt-Universität zu Berlin, Berlin, Germany; 4 Programme area Systems Rheumatology, Deutsches Rheuma-Forschungszentrum Berlin, ein Institut der Leibniz-Gemeinschaft, Berlin, Germany; 5 Department of Paediatric Pneumology, Allergology and Neonatology, Children’s Hospital, Hannover Medical School, Hannover, Germany; 6 German Centre for Paediatric and Adolescent Rheumatology, Garmisch-Partenkirchen, Germany; 7 Pediatric Rheumatology, Children’s Hospital St. Marien, Landshut, Germany; 8 Department of Pediatric Rheumatology, Asklepios Kinderklinik Sankt Augustin, Sankt Augustin, Germany; 9 Department of Paediatric and Adolescent Medicine, University Hospital of Cologne, Cologne, Germany; 10 Division of Pediatric Infectious Diseases and Rheumatology, Department of Pediatrics and Adolescent Medicine, University Medical Center, Freiburg, Germany; 11 Department of Pediatrics and Adolescent Medicine, Pediatric Rheumatology, Eltern-Kind-Zentrum Prof. Hess, Klinikum Bremen-Mitte, Bremen, Germany; 12 Clinic of Paediatric and Adolescent Rheumatology, Northwest German Centre for Rheumatology, St. Josef-Stift Sendenhorst, Sendenhorst, Germany; 13 Medizinische Fakultät, Universität Halle-Wittenberg, Halle, Germany; 14 Department of Paediatrics, Helios Klinik Berlin-Buch, Berlin, Germany; 15 Children’s Hospital, Vivantes Klinikum Friedrichshain, Berlin, Germany; 16 Department of Pediatric Oncology, Hematology and Clinical Immunology, Division of Pediatric Rheumatology, University Hospital, Medical Faculty, Heinrich Heine University, Düsseldorf, Germany; 17 Hamburg Centre for Paediatric and Adolescent Rheumatology, Schön Klinik Hamburg Eilbek, Hamburg, Germany; 18 Department of Pediatrics and Neonatology, Division of Pediatric Rheumatology, University Hospital of Marburg and Gießen, Gießen, Germany; 19 Department of Rheumatology & Immunology, Dr. von Hauner Children’s Hospital, Ludwig-Maximilians-University, Munich, Germany

**Keywords:** Physical activity, Juvenile idiopathic arthritis, Children, Adolescents, Controls

## Abstract

**Background:**

Physical active lifestyles are essential throughout growth and maturation and may offer potential preventive and therapeutic benefit in patients with juvenile idiopathic arthritis (JIA). Insufficient physical activity (PA), in contrast, can lead to aggravation of disease-related symptoms. This study aimed to i) examine PA levels in children and adolescents with JIA compared to general population controls and ii) investigate correlates of pronounced physical inactivity in order to identify risk groups for sedentary behaviour.

**Methods:**

Data from children and adolescents with JIA and population controls aged 3 to 17 years documented in the National Pediatric Rheumatologic Database (NPRD) and the German Health Interview and Examination Survey for Children and Adolescents (KiGGS) were used. Self-reported PA was collected from parents/guardians of children up to 11 years of age or adolescents 12 years of age and older. To compare PA-related data, age- and sex-specific pairwise analyses were conducted considering NPRD/KiGGS participants' data from 2017. Correlates of physical inactivity among patients were identified using a linear regression model.

**Results:**

Data of 6,297 matched-pairs (mean age 11.2 ± 4.2 years, female 67%, patients’ disease duration 4.5 ± 3.7 years, persistent oligoarthritis 43%) were available for evaluation. Almost 36% of patients aged 3–17 years (vs. 20% of controls) achieved the WHO recommended amount of PA, while PA steadily decreased with age (18% of patients aged ≥ 12 years) and varied between JIA categories. Female adolescents and patients with enthesitis-related arthritis were least likely to achieve the minimum recommended level of PA. Physical inactivity was associated with female sex, higher age at disease onset, longer disease duration, more functional disability (C-HAQ) and higher disease activity (cJADAS-10).

**Conclusions:**

Depending on JIA category, children and adolescents with JIA were similarly or even more likely to achieve the WHO recommended minimum level of PA compared to general population controls. However, since a large proportion of young JIA patients appear to be insufficiently physically active, engagement in targeted efforts to promote PA is urgently needed.

**Supplementary Information:**

The online version contains supplementary material available at 10.1186/s12969-024-00976-2.

## Background

Juvenile idiopathic arthritis (JIA) is a heterogeneous group of inflammatory diseases of unknown origin, with onset before the age of 16 years. JIA is the most common chronic rheumatic disease affecting the pediatric population, with prevalence numbers ranging from 2 to 20 per 100,000 in European countries and up to 168 per 100,000 in Germany [1, 2]. Characterized by an overproduction of inflammatory cytokines at the joint level, JIA leads to a chronic inflammatory state accompanying joint pain, fatigue, stiffness and movement restrictions [3]. These symptoms and certain medical treatments for managing JIA place children at increased risk of sub-optimal bone mineralization and osteoporosis, malnutrition, muscle weakness, mobility impairments, and limitations in activities of daily living, such as playing [3–7].

Promoting healthy lifestyles including physical activity (PA) is an intuitively attractive strategy to alleviate several disease-related symptoms such as low aerobic fitness, pain, fatigue, muscle weakness and poor health-related quality of life [5, 8–10]. Although the propensity of young patients with JIA to be physically hypoactive can be detrimental to general disease symptoms and function [11], previous studies have shown that they tend to be less physically active [12, 13], spend more time in sedentary behaviour and less time in WHO [14] recommended health-enhancing PA of moderate-to-vigorous intensity compared to healthy controls [15].

However, previous data on PA levels among children and adolescents with JIA were frequently based on small samples, considered only certain JIA categories and age ranges, and did not provide data of representative controls, thus leading to inconsistent conclusions. There is currently a lack of reliable information on clinical barriers to PA in this population, knowledge of which, however, is a prerequisite for promoting active lifestyles and deriving appropriate interventions.

The aim of this study was to provide information on PA levels in a very large JIA cohort compared to representative population controls. We examined the extent of pronounced physical inactivity and its potential correlates in order to identify clinical barriers and risk groups for a physically inactive lifestyle.

## Methods

### Patients

Patients in this multicentre observational study were included within the framework of the National Pediatric Rheumatologic Database (NPRD). The nationwide NPRD captures a broad spectrum of juvenile rheumatic diseases and annually collects data on disease phenomena and outcome measures using standardized physician and patient questionnaires. According to estimates, the number of JIA cases recorded each year corresponds to approximately 50% of all expected cases in Germany. Further details on this representative database, containing sociodemographic and clinical characteristics as well as treatment assignments, can be found in Minden et al. [16–18]. The study was approved by the ethics committee of the Charité – Universitätsmedizin Berlin (EA1/044/07).

Inclusion criteria for the analyses in the present study were as follows: 1) diagnosis of JIA according to the International League of Associations for Rheumatology (ILAR) criteria [3], 2) documentation in the database in 2017, and 3) age at documentation in 2017 between 3 and 17 years. Age range and year 2017 were chosen for comparability reasons with the reference population documented within KiGGS [19].

### Controls

Children and adolescents who participated in the nationwide German Health Interview and Examination Survey for Children and Adolescents (KiGGS) served as controls. KiGGS as part of the health-monitoring programme undertaken at the Robert Koch-Institute includes repeated cross-sectional surveys of children and adolescents aged between 0 and 17 years that are representative of the German population [19]. Participants were randomly chosen from 167 registration offices and invited for interviews, physical examinations, and laboratory tests. The study design and sampling procedure are described in detail by Kurth et al. [20].

### Physical activity

Physical activity was self-reported (age group 12–17 years) or provided by parents or legal guardians (3–11 age group). This took place either in clinical setting or in a home environment immediately before or after the routine consultation. Patients and parents/legal guardians were asked, ‘On how many days of a normal week are you/is your child physically active for at least 60 min on a single day?’ The eight answer categories ranged from ‘On no day’ to ‘On seven days’. Respondents did not receive any details or examples when completing the question.

Based on this data, and according to the methodology used in the reference population [20], the question estimated whether children and adolescents met the WHO recommended ‘at least 60 min of moderate to vigorous PA per day’. Also, in line with the reference population, an indicator for ‘physical inactivity’ was created for those who are physically active for at least 60 min per day on less than two days per week.

A comparison of self-reported PA between patients of the NPRD and peers of the KiGGS sample [19] was performed for 3–17-year-olds documented/surveyed in 2017.

Analyses of cross-sectional associations between physical inactivity, sociodemographic and clinical parameters were conducted for the total JIA cohort as well as explicitly for patients from the age of 12. The age limit of 12 years and older was chosen because from this age onwards, NPRD patients have to fill in the questionnaire independently without any parental evaluation.

### Clinical data

Sociodemographic data reported by the pediatric rheumatologists included patient age, sex, diagnosis, age at disease onset, current height, and weight. BMI was calculated as the weight in kilograms divided by the height in metres squared. Underweight (BMI < 10th), normal weight (BMI ≥ 10th—≤ 90th), overweight (BMI > 90th) and obesity (BMI > 97th) were defined according to age- and sex-specific percentiles used in the German reference system [21]. Additionally, the rheumatologist assessed patient's disease activity (physician's global assessment, PGA) on a numerical rating scale (NRS; from 0 = no disease activity to 10 = very severe disease activity). Regarding drug therapy, treatment with glucocorticoids (GCs) as well as conventional synthetic (csDMARDs) and biologic (bDMARDs) disease-modifying antirheumatic drugs within the last 12 months were recorded. Treatment with systemic GCs included low-dose (< 0.2 mg/kg body weight/day), high-dose (≥ 0.2 mg/kg/day), and intravenous pulse therapy categories. In addition, intra-articular GC injections were reported.

Patients aged ≥ 12 years and parents of patients aged < 12 years reported on functional ability using the German version of the Childhood Health Assessment Questionnaire (C-HAQ) [22]. The resulting disability index can range from 0 to 3, whereby a value of zero indicates no functional disability and higher scores indicate light, moderate, or severe level of disability. Reported parental education ranged from ‘no qualification’ (primary education), ‘secondary education’ and ‘higher education (Graduation)’. Patient-/parent-reported outcomes on an NRS (0–10) included an assessment of pain, fatigue, coping and overall well-being.

Based on physician- and patient-reported data, disease activity was assessed with the clinical Juvenile Arthritis Disease Activity Score in 10 joints (cJADAS-10) [23]. The cJADAS-10 considers the number of joints with active disease, physicians' global assessment of disease activity and patients' rating on well-being. In accordance with Trincianti et al. [24], the cJADAS-10 cut-off for classification of minimal disease activity was 4 for oligoarticular JIA and 5 for polyarticular JIA.

### Statistical analyses

Categorical variables were reported by numbers and percentages, whereas continuous variables were reported by means and standard deviations (95%-confidence interval). An age- and sex-matched sample of children and adolescents aged 3 to 17 years from the KiGGS study [19] was used to compare PA levels of JIA patients with the general German population. Levels of PA were determined for 2017 in the NPRD total cohort and in the control group, while frequencies were analysed for various subgroups (age groups, sexes, and JIA categories). The association between sociodemographic as well as clinical parameters and physical inactivity is described by odds ratios (ORs) with 95%-confidence intervals. All *p*-values less than 0.05 were considered to be statistically significant. As part of a sensitivity analysis, we also determined patients’ PA levels based on data from 2019 (pre-pandemic) to 2022 (during pandemic). Statistical analyses were performed using SAS 9.3 (SAS Institute Inc., Cary, NC, USA).

## Results

For the year 2017, data from 6,297 age- and sex-matched pairs of 3–17 year olds could be considered. Approximately two-thirds of the sample were female. The number of pairs varied between age groups (Table 1).

**Table 1:** Sociodemographic and clinical characteristics of JIA patients recorded in the year 2017

	**Total**	**Age group ≤ 5 years**	**Age group 6 to 11 years**	**Age group ≥ 12 years**
No. of patients	6,297	816	2,159	3,322
Age, years	11.2 (4.2)	3.9 (1.0)	8.7 (1.7)	14.6 (1.7)
Female, no. (%)	4,248 (67.5)	618 (75.7)	1,445 (66.9)	2,185 (65.8)
BMI, kg/m2, no. (%)				
SDS	-0.02 (2.12)	-0.09 (1.14)	-0.09 (2.11)	0.04 (2.30)
underweight	736 (11.9)	86 (10.8)	256 (12.0)	394 (12.1)
normal weight	4,533 (73.3)	646 (81.3)	1,583 (74.3)	2,304 (70.7)
overweight	563 (9.1)	41 (5.2)	182 (8.5)	340 (10.4)
obesity	352 (5.7)	22 (2.8)	110 (5.2)	220 (6.8)
Disease duration, years	4.5 ± 3.7	1.5 ± 1.1	4.0 ± 2.6	5.7 ± 4.3
Age at disease onset, years	6.4 ± 4.3	2.3 ± 1.1	4.6 ± 2.7	8.8 ± 4.4
JIA category, no. (%)				
RF-positive polyarthritis	129 (2.0)	4 (0.5)	19 (0.9)	106 (3.2)
RF-negative polyarthritis	1,277 (20.3)	166 (20.3)	449 (20.8)	662 (19.9)
Systemic JIA	242 (3.8)	34 (4.1)	86 (4.0)	122 (3.6)
Persistent oligoarthritis	2,698 (42.9)	505 (61.9)	1,074 (49.7)	1,119 (33.7)
Extended oligoarthritis	721 (11.5)	66 (8.1)	259 (12.0)	396 (11.9)
Psoriatic arthritis	296 (4.7)	21 (2.6)	78 (3.6)	197 (5.9)
Enthesitis-related arthritis	739 (11.7)	3 (0.4)	127 (5.9)	609 (18.3)
Unclassified JIA	195 (3.1)	17 (2.1)	67 (3.1)	111 (3.3)
Uveitis reported, no. (%)	750 (13.2)	82 (10.6)	321 (16.3)	347 (11.9)
PGA score	1.3 (1.8)	1.7 (2.1)	1.1 (1.7)	1.3 (1.9)
cJADAS-10	4.0 (4.7)	4.5 (4.9)	3.2 (4.1)	4.4 (4.9)
Number of joints with active disease	1.0 (2.7)	1.2 (2.6)	0.7 (2.1)	1.1 (3.0)
C-HAQ total score	0.2 (0.4)	0.3 (0.5)	0.2 (0.4)	0.2 (0.4)
Patient-reported pain^a^	1.9 (2.5)	1.6 (2.4)	1.4 (2.2)	2.2 (2.7)
Patient-reported overall well-being^a^	1.8 (2.2)	1.7 (2.1)	1.5 (1.9)	2.1 (2.3)
Patient-reported fatigue^a^	1.5 (2.4)	1.4 (2.2)	1.1 (2.0)	1.7 (2.6)
Patient's global assessement^a^	2.4 (2.9)	2.9 (3.3)	2.3 (3.1)	2.3 (2.7)
Treatment in past 12 months, no. (%)				
NSAIDs	1,991 (37.5)	317 (46.6)	613 (33.7)	1,061 (37.9)
Intra-articular GCs	677 (12.8)	149 (21.9)	235 (12.9)	293 (10.5)
Low dose GCs (< 0.2 mg/kg)	234 (4.4)	33 (4.9)	66 (3.6)	135 (4.8)
High dose GCs or pulse therapy	320 (5.0)	70 (10.3)	95 (5.2)	155 (5.5)
Any conventional synthetic DMARD	2,571 (47.8)	346 (52.0)	962 (51.8)	1,263 (44.3)
Any biologic DMARD	1,312 (24.4)	82 (12.3)	393 (21.2)	837 (29.3)
Highest parental education				
No qualification (primary education)	179 (5.2)	25 (3.4)	119 (6.1)	35 (4.7)
Secondary education	1,780 (51.9)	351 (47.3)	1,017 (52.4)	412 (54.9)
Higher education (Graduation)	1,473 (42.9)	366 (49.3)	804 (41.4)	303 (40.4)

*JIA* juvenile idiopathic arthritis, *BMI* body mass index, *SDS* Standard Deviation Score, *RF* rheumatoid factor, *PGA* physician’s global assessment, *cJADAS-10* 10-joint clinical Juvenile Arthritis Disease Activity Score, *C-HAQ* Childhood Health Assessment Questionnaire, *NSAID* non-steroidal anti-inflammatory drug, *GC* glucocorticoid, *DMARD* disease-modifying antirheumatic drug

^a^Assessed on a numerical rating scale (maximum score 10)

### Sociodemographic and clinical characteristics

Relevant differences in patient characteristics, involving sex ratio, weight status, disease onset and disease duration, were found across age groups. The mean age of patients at disease onset was 2.3 ± 1.1 years in the age group ≤ 5 years and 8.8 ± 4.4 years in patients aged 12 and above. Persistent oligoarthritis was most common in all age groups but decreased steadily with age. While the proportion of patients treated with bDMARDs increased with age (from approximately 12% in the age group ≤ 5 years to 29% in the age group ≥ 12 years), csDMARDs were used less frequently with age. An age-related decrease was also observed in the use of intra-articular GCs (from 21.9% in the age group ≤ 5 years to 10.5% in the age group ≥ 12 years). More details of clinical data are shown in Table 1.

### Comparison of physical activity between JIA patients and controls

**Fig. 1:**
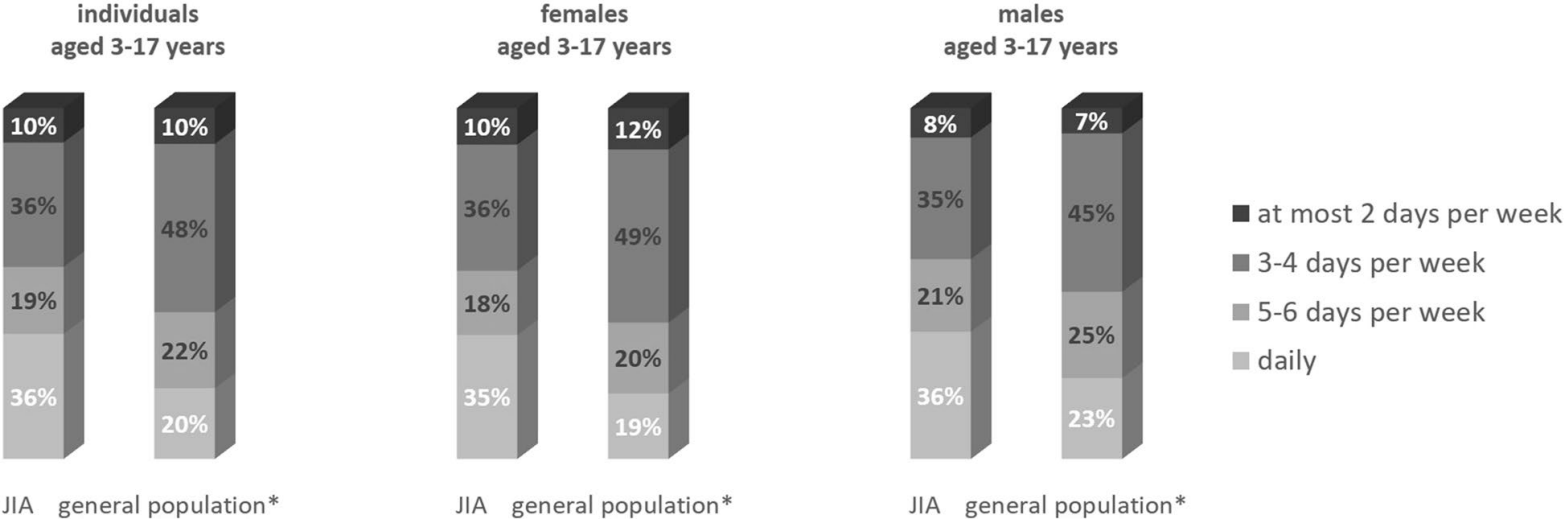
Number of weekdays with at least 60 min of physical activity. *age- and sex-matched sample

In 2017, 35.7% of children and adolescents with JIA and 20.2% of controls met the WHO recommended minimum level of PA (Fig. 1). For the (pre-)pandemic period 2019–2022, patients' PA levels were constant, with proportions of those adhering to PA guidelines ranging from 37–40% (results not shown). The likelihood of achieving PA recommendations in 2017 was higher in patients than in controls across all age groups. However, differences between patients and controls decreased with increasing age (age group 3–6: ∆ 30%, age group 7–10: ∆ 23%, age group 11–13: ∆ 12%, age group 14–17: ∆ 6%).

**Fig. 2:**
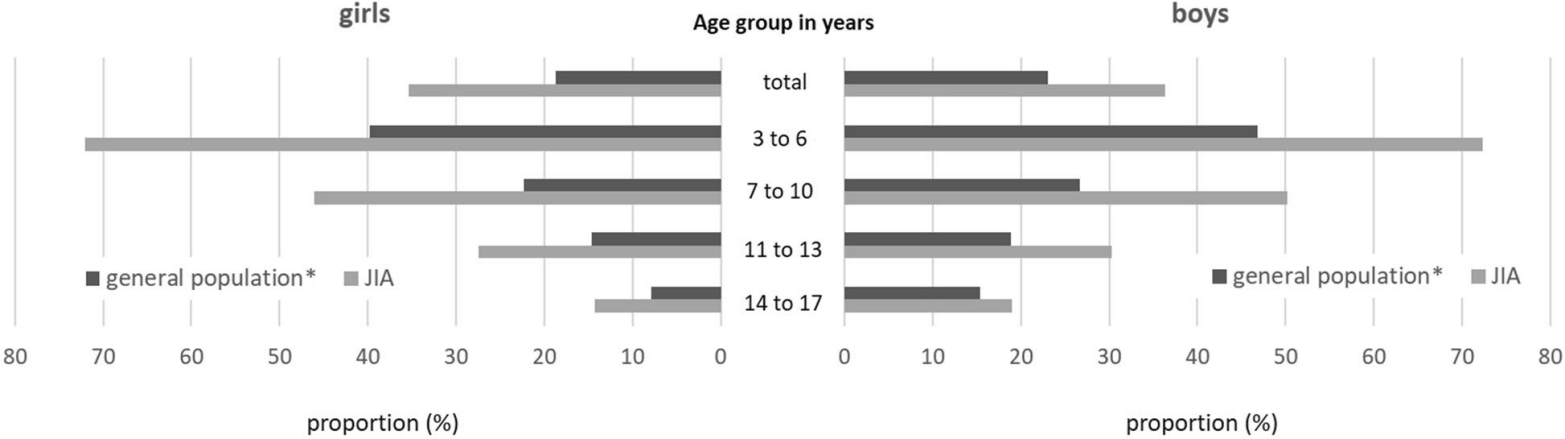
Prevalence of at least 60 min of physical activity per day by age group and sex ("WHO recommendation achieved"). *age- and sex-matched sample

**Fig. 3:**
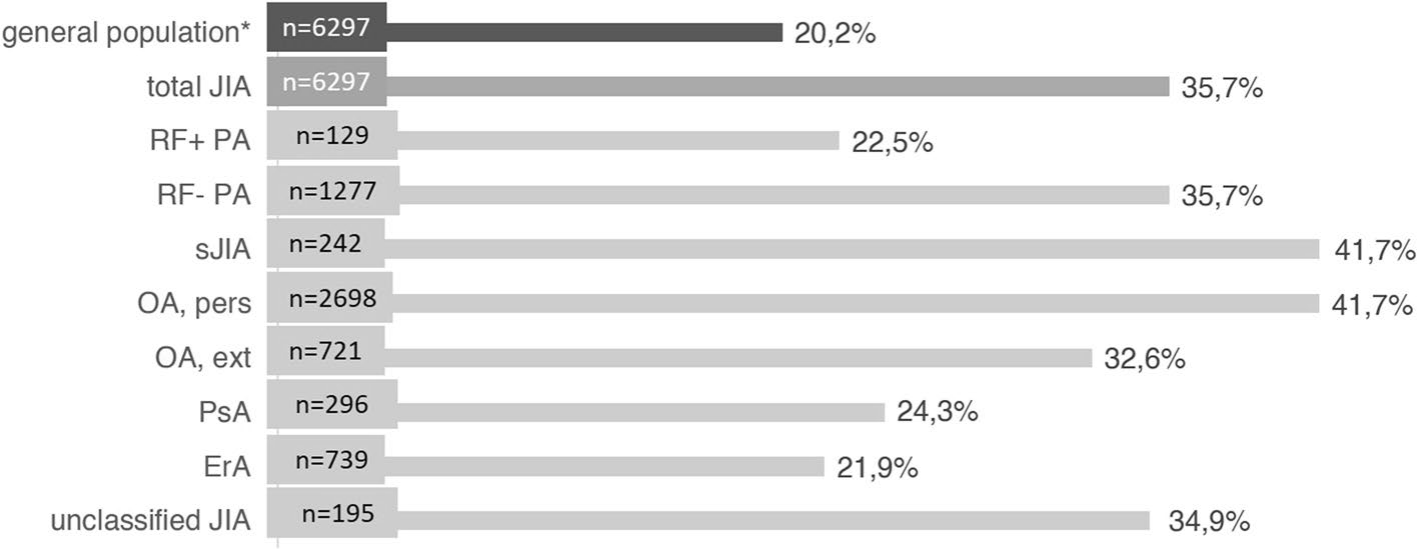
Prevalence of at least 60 min of physical activity per day according to JIA category ("WHO recommendation achieved"). *age- and sex-matched sample

In the general population, girls were less likely to reach the WHO recommendation on PA than boys in all age groups. With exception of 3–6-year-olds (72.1% vs. 72.2%), this was also observed in patients (Fig. 2). Patients with systemic arthritis (41.7%) and persistent oligoarthritis (41.7%) achieved the recommended level of PA most frequently (Fig. 3).

### Comparison of physical inactivity between JIA patients and controls

The proportion of physically inactive individuals (PA < 2/week for at least 60 min) was comparable among patients (9.8%) and controls (10.3%). However, girls were slightly more likely to be physically inactive than boys in both patients (10.4% vs 8.4%) and controls (11.8% vs 7.2%).

**Fig. 4:**
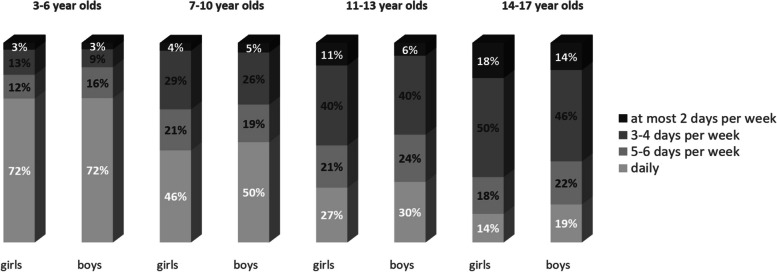
Number of weekdays with at least 60 min of physical activity in female and male patients with JIA

The largest proportion of physically inactive patients (16.2%) and controls (16.8%) was registered in individuals aged 14–17 years (Fig. 4). Among them, female patients (17.6% vs. 13.6%) and female controls (20.1% vs. 10.6%) were significantly more likely to be physically inactive than their male counterparts. The largest proportion of physically inactive individuals was registered among patients with rheumatoid factor-positive polyarthritis (14.0%) and enthesitis-related arthritis (13.1%).

### Physical inactivity and its cross-sectional correlates

In total, 9.8% (*n* = 614) of patients aged 3–17 years reported being physically active less than twice per week for at least 60 min. Among them, 80.3% (*n* = 493) were 12 years or older. While 23.3% had a cJADAS-10 score ≤ 1, 45.3% had a CHAQ of 0. In addition, 42.8% rated their pain intensity, 35.6% their general health, and 52.2% their fatigue as ≤ 1. While male sex (OR 0.72 [0.54–0.95]; *p* = 0.021) was associated with a lower likelihood of physical inactivity, increasing disease duration (OR 1.38 [1.32–1.45]; *p* =  < 0.001), disease age (OR 1.37 [1.32–1.42]; *p* =  < 0.001), disease activity (OR 1.07 [1.04–1.10]; *p* =  < 0.001), and functional disability (OR 2.19 [1.61–2.98]; *p* =  < 0.001) increased the likelihood of physical inactivity in patients aged 3–17 years. Multivariable correlates of physical inactivity in adolescents aged 12 and above are shown in Table 2.

**Table 2:** Multivariable correlates of physical inactivity in patients recorded in 2017

	**Age group 3–17 years (** ***n*** ** = 614)**			**Age group ≥ 12 years (** ***n*** ** = 493)**		
Variable	OR	95%-CI	*P*	OR	95%-CI	*P*
Male sex	**0.72**	**0.54–0.95**	**0.021**	**0.61**	**0.43–0.85**	**0.004**
Disease duration	**1.38**	**1.32–1.45**	** < 0.001**	**1.28**	**1.17–1.42**	** < 0.001**
Age at JIA onset	**1.37**	**1.32–1.42**	** < 0.001**	**1.26**	**1.15–1.38**	** < 0.001**
cJADAS-10	**1.07**	**1.04–1.10**	** < 0.001**	**1.08**	**1.04–1.12**	** < 0.001**
C-HAQ	**2.19**	**1.61–2.98**	** < 0.001**	**1.68**	**1.11–2.54**	**0.015**
csDMARD	1.25	0.96–1.62	0.094	1.10	0.80–1.51	0.545
bDMARD	0.77	0.57–1.04	0.093	0.74	0.52–1.05	0.090

Physical inactivity, physical activity < 2 times/week for at least 60 min

*OR* Odds ratio, *95%-CI* 95%-confidence interval, *JIA* juvenile idiopathic arthritis, *JADAS-10* 10-joint Juvenile Arthritis Disease Activity Score, *C-HAQ* Childhood Health Assessment Questionnaire, *csDMARD* conventional synthetic disease-modifying antirheumatic drug, *bDMARD* biologic disease-modifying antirheumatic drug

## Discussion

The results of this nationwide representative study in children and adolescents with JIA extend the current literature by providing information on self-reported PA levels and their cross-sectional correlates. In 2017, patients with JIA achieved the WHO-recommended minimum level of PA comparable to or even more frequently than the general population, depending on JIA category. However, a significant decrease in PA level was observed with increasing age in both patients and controls.

At least eight in ten adolescents with JIA did not achieve the minimum level of physical activity, while one to two in ten (15%) were even considered highly physically inactive. The proportion of physical inactivity was comparable to general population controls. In patients, female sex, longer disease duration, older age at JIA onset, more functional limitations and higher disease activity were associated with physical inactivity.

Our data are based on one of the most extensive analyses of PA levels and its correlates conducted in pediatric JIA patients to date.

We found that the overall group of children and adolescents with JIA had higher PA levels on average than the German general population, although PA levels between patients and controls converged with age. Thus, our results differ from recent reports showing that young patients with JIA have lower PA levels than healthy peers [13, 25]. Other studies providing information on PA state that overall PA levels in JIA patients diagnosed in the era of biologics were similar to controls [15, 26]. In this context, Sherman et al. [26] postulate that this lack of difference may be due to clinical remission following an early, aggressive treat-to-target strategy. However, it should be noted that these studies reported PA levels only for the whole group, probably due to small sample sizes, narrow age ranges and underrepresentation of certain JIA categories (particularly oligoarthritis). In contrast, the distribution of categories in our cohort differs only slightly from that previously reported for Europe [27]. As we found significant variations in PA levels between JIA categories in our large, representative sample, this may partly explain discrepancies with overall PA reported in other studies. However, it is also conceivable that other characteristics not recorded in the study populations, such as socioeconomic status or cultural characteristics, may complicate comparability.

Patients with systemic arthritis and persistent oligoarthritis were significantly more likely to achieve the recommended PA level than age- and sex-matched controls. Consistent with previous studies, patients with enthesitis-related arthritis [28] and rheumatoid factor-positive polyarthritis [29] reported lowest PA levels. This could be explained on the one hand by a higher disease burden due to a higher number of active joints and on the other hand by the affection of the axial skeleton and a higher presence of enthesopathy with increasing pain in the lower extremities during all weight-bearing activities. In addition, the location of arthritis in the lower limbs, particularly in hip and knee joints, appears to be important as these affect walking endurance, cycling performance, function and quality of life [30]. Further, the onset of disease in enthesitis-related arthritis and rheumatoid factor-positive polyarthritis often occurs during puberty, an age phase generally associated with lower PA levels even in healthy individuals.

This age phase is usually accompanied by increasing independence from parents, changing life circumstances (e.g. employment) and many competing leisure activities (e.g. electronic media use) [31, 32]. Therefore, psychosocial and environmental changes are disease-independent factors that may have contributed to lower PA levels among adolescent patients and population controls.

Regardless, it should be noted that the WHO guidelines for PA of at least 60 min per day are only a minimum recommendation and that any PA beyond this may have additional health benefits [14]. This is reflected in the national guidelines for PA promotion in Germany, recommending at least 180 min of daily PA for children of kindergarten age and 90 min of daily PA for children and adolescents in school age [33].

In our study, one in ten children and adolescents aged 3 to 17 years was classified as highly physically inactive, with a comparable proportion in patients and controls. With slight sex differences to the disadvantage of girls, the proportion of physically inactive individuals increased during puberty, while the proportion of those who reached the recommended minimum level of PA decreased simultaneously (two out of ten adolescents). The observed decline in PA with age has already been described in several studies in healthy children and adolescents and is associated with an average decrease of 6 min per day per year of moderate to vigorous PA [34].

We identified as correlates of physical inactivity longer disease duration, older age at disease onset, higher disease activity and more functional limitations. Thus, our results are comparable to previous findings reporting associations between PA level, disease duration and disease activity [35, 36]. Although non-pharmacological, physical activity-based therapies are increasingly becoming part of the JIA treatment protocol [37], previous results have suggested that physical inactivity is not only the result of disease-related impairments [17, 25]. This observation can also be confirmed by the results within the framework of our study. Considering existing cut-off values [24], 51% of physically inactive adolescents (≥ 12 years) with oligoarthritis and 38% with polyarthritis were in a state of minimal disease activity. Although we cannot rule out that other clinical factors are responsible for physical inactivity in some patients, this finding highlights the importance of applying behavior change techniques to adolescents. As studies indicate that measures to contain the COVID-19 pandemic have had negative impacts on PA among European children and adolescents [38], this becomes all the more important. However, negative effects of pandemic-related restrictions were not reflected in the level of self-reported PA in our patients, at least until 2022. In patients with JIA, chronic inflammation with persisting systemic circulating inflammatory proteins constitute a risk for early vascular damage, with general physical inactivity further increasing this risk.

The strength of our study includes prospective cross-sectional evaluations using an observational cohort study with representative data on clinical characteristics as well as treatment assignments of children and adolescents with JIA in Germany. According to estimates, the number of JIA patients included corresponds to almost 45% of all expected JIA cases in Germany [39]. PA levels were assessed analogously to the methodology used in the reference population [19], considering clinically relevant parameters as well as general and disease-specific instruments.

Nevertheless, our results must be interpreted with caution given several possible limitations. We did not capture PA intensity and are therefore unable to accurately comment on whether this cohort of children and adolescents is meeting current PA recommendations of 60 min of moderate to vigorous PA per day. As respondents were not given any details or examples when completing the question on PA, it cannot be ruled out that PA was sometimes interpreted differently by individuals. Furthermore, the question on PA was based on self-reports. Although, most PA self-reports are suitable for classifying subjects according to their PA levels and are commonly used in pediatric and population-based research [40–43], it is well recognized that their accuracy is limited and can lead to over- or under-reporting for a variety of reasons. In our JIA cohort, socially desirable responding in the presence of hospital staff, for example, might be one possible reason. Moreover, parent-reported outcomes may have led to an overestimation of PA levels in the younger cohort (patients aged < 12 years). Based on the parental education level recorded, most of our patients probably have a medium to high socioeconomic status (SES). Since the NPRD does not ask about income, we were not able to relate SES to PA. Finally, we were not able to examine health-related quality of life, which is known to be associated with PA levels in many patient and healthy populations.

## Conclusions

In conclusion, children and adolescents with JIA are similarly or even more likely to achieve the WHO recommended minimum level of PA compared to general population controls, however, an overwhelming proportion is insufficiently physically active, partly despite satisfactory control of inflammation. As adolescent patients and patients with enthesitis-related arthritis, rheumatoid factor-positive polyarthritis as well as psoriatic arthritis are particularly vulnerable to physical inactivity, PA should be promoted specifically for these subgroups and for all patients with symptoms allowing PA. To achieve this, all those working with children and young people with JIA need to encourage them to engage in physical activities appropriate to their symptoms, lifestyle and fitness level.

In order to promote activities of daily life and to implement adequate interventions, JADAS and CHAQ should be controlled. To clarify both the safety of PA and the health risks associated with physical inactivity, efforts are needed to improve the quality of information provided for parents, health professionals, teachers and patients. Future studies should provide a better understanding of how socioeconomic status, health-related quality of life, and other patient-reported outcomes (e.g. mental health, sleep quality) relate to PA behaviour in JIA.

### Supplementary Information

**Supplementary Material 1.**

## Data Availability

The datasets used and/or analysed during the current study are available from the corresponding author on reasonable request.

## Abbreviations

bDMARDs: Biological disease-modifying antirheumatic drugs
BMI: Body Mass Index
C-HAQ: Childhood Health Assessment Questionnaire
cJADAS-10: Clinical Juvenile Arthritis Disease Activity Score in 10 joints
csDMARDs: Conventional synthetic disease-modifying antirheumatic drugs
GCs: Glucocorticoids
ILAR: International League of Associations for Rheumatology
JIA: Juvenile idiopathic arthritis
KiGGS: German Health Interview and Examination Survey for Children and Adolescents
NPRD: National Pediatric Rheumatologic Database
NRS: Numerical Rating Scale
PA: Physical activity
PGA: Physician's global assessment
ORs: Odds ratios
WHO: World Health Organization
