# Supplementary material for: Level and correlates of physical activity among children and adolescents with juvenile idiopathic arthritis compared to controls: results from a German nationwide prospective observational cohort study
Source: Pediatr Rheumatol Online J. 2024 Mar 20;22:39. doi: 10.1186/s12969-024-00976-2 (PMC10953124; doi:10.1186/s12969-024-00976-2)
Supplement: Supplementary file 1 — Supplementary Material 1. [file 12969_2024_976_MOESM1_ESM.docx]

Tomas Berger, Vestische Kinder- und Jugendklinik Datteln, Rheumatologie/Immunologie, Datteln; Rainer Berendes, Kinderklinik St. Marien, Landshut; Michael Borte, Städtisches Klinikum St. Georg, Klinik für Kinder- und Jugendmedizin, Leipzig; Jürgen Brunner, Medizinische Universität Innsbruck, Kinder- und Jugendheilkunde, Innsbruck; Frank Dressler, Medizinische Hochschule Hannover, Kinderklinik, Hannover; Ivan Foeldvari, Hamburger Zentrum für Kinder- und Jugendrheumatologie, Schwerpunktpraxis am Klinikum Eilbek, Hamburg; Dirk Föll, Universitätsklinik Münster, Klinik für Pädiatrische Rheumatologie und Immunologie, Münster; Matthias Galiano, Universitätsklinikum Erlangen, Kinder- und Jugendklinik, Erlangen; Hermann Girschick, Vivantes Klinikum Friedrichshain, Berlin; Kinderarzt Jörn-Lorenz Gröbel, Paderborn; Jürgen Grulich-Henn, Universitätsklinikum Heidelberg, Zentrum für Kinderund Jugendmedizin - Kinderheilkunde I, Heidelberg; Johannes-Peter Haas, Deutsches Zentrum für Kinderund Jugendrheumatologie, Garmisch-Partenkirchen; Maria Haller, Kinderarztpraxis, Gundelfngen; Georg Heubner, Städtisches Klinikum Dresden-Neustadt, Klinik für Kinder- und Jugendmedizin, Dresden; Nadja Hofmann, Sozialstiftung Bamberg, Klinik für Kinder und Jugendliche, Bamberg; Anette Holl-Wieden, Universitätsklinikum Würzburg, Kinderklinik und Poliklinik, Würzburg; Gerd Hornef, Asklepios Kinderklinik St. Augustin, Zentrum für Allgemeine Pädiatrie und Neonatologie, Sankt Augustin; Anton Hospach, Zentrum für Pädiatrie, Olgahospital, Klinikum Stuttgart; Regina Hühn, Martin-Luther-Universität Halle-Wittenberg, Halle (Saale); Markus Hufnagel, Zentrum für Kinder- und Jugendmedizin, Universitätsklinikum, Freiburg; Ales Janda, Universitätsklinikum Ulm, Klinik für Kinder- und Jugendmedizin, Ulm; Annette Jansson, Dr.-von-Haunersches Kinderspital der LMU, Kinderklinik und Kinderpoliklinik, München; Kinderarztpraxis Annette Jentzsch, Vechelde; Tilmann Kallinich, Universitätsmedizin Berlin - Charité, Campus Virchow-Klinikum, Otto-Heubner-Centrum für Kinder- und Jugendmedizin, Berlin; Tomas Keller, Josefinum Krankenhaus, Klinik für Kinder und Jugendliche, Augsburg; Jasmin B. Kümmerle-Deschner, Universitätsklinikum Tübingen, Zentrum für Kinder- und Jugendrheumatologie, Tübingen; Kinderarztpraxis Rolf-Michael Küster, Wedel; Elke Lainka, Universitäts-Kinderklinik Essen, Zentrum für Kinderund Jugendmedizin, Essen; Georg Leipold, Gemeinschaftspraxis Kinder- und Jugendärzte, Regensburg; Jan Maier, Kinderarztpraxis, Leinfelden-Echterdingen; Kristina Mathony, Städtisches Klinikum Dessau, Klinik für Kinder- und Jugendmedizin, Dessau; Almut Meyer-Bahlburg, Universitätsmedizin Greifswald, KöR, Abt. Allgemeine Pädiatrie, Greifswald; Kirsten Minden, Universitätsmedizin Berlin - Charité, Campus Virchow-Klinikum, Otto-Heubner-Centrum für Kinder- und Jugendmedizin, Berlin; Kirsten Mönkemöller, Kinderkrankenhaus der Stadt Köln, Kinder- und Jugendmedizin, Köln; Tim Niehues, Helios Klinikum Krefeld, Pädiatrische Institutsambulanz, Krefeld; Kinderarztpraxis Nimtz-Talaska, Frankfurt/Oder; Nils Onken, Kinderarztpraxis, Lüneburg;

Prassad T. Oommen, Med. Einrichtungen der Heinrich-Heine-Universität, Zentrum für Kinder -und Jugendmedizin, Düsseldorf; Claudia Präger, Diakonie-Klinikum Schwäbisch Hall, Kinderklinik, Schwäbisch Hall; Jürgen Quietzsch, DRK Krankenhaus Lichtenstein, Klinik für Kinder- und Jugendmedizin, Lichtenstein; Christiane Reiser, Kinderklinik, Landeskrankenhaus Bregenz, Bregenz; Christoph Rietschel, Clementine Kinderhospital, Klinik für Kinder- und Jugendmedizin, Frankfurt; Kinderrheuma Praxis Betina Rogalski, Bensheim; Michael Rühlmann, Kinderarztpraxis, Göttingen; Peggy Rühmer, Helios Vogtland-Klinikum Plauen, Fachambulanz der Klinik für Kinder- und Jugendmedizin, Plauen; Axel Sauerbrey, Helios Klinikum Erfurt, Klinik für Kinder- und Jugendmedizin, Erfurt; Volker Schuster, Universitätsklinik und Poliklinik für Kinder und Jugendliche, Rheumaambulanz, Leipzig; Catharina Schütz, Universitätsklinikum Carl Gustav Carus, Klinik und Poliklinik für Kinder- und Jugendmedizin, Dresden; Anja Sonnenschein, Johannes Gutenberg-Universität Mainz, Zentrum für Kinder- und Jugendmedizin, Mainz; Claudia Stollbrink, Universitätsklinikum Aachen; Kinderklinik RWTH Aachen, Klinik für Kinder- und Jugendmedizin, Aachen; Ralf Trauzeddel, Helios Klinikum Berlin-Buch, Klinik für Kinderund Jugendmedizin, Berlin; Philipp von Bismarck, Universitätsklinikum Schleswig Holstein - Campus Kiel, Klinik für Kinder- und Jugendmedizin, Kiel; Frank Weller-Heinemann, Klinikum Bremen Mitte – Professor Hess-Kinderklinik, Zentrum für Kinder- und Jugendrheumatologie, Bremen; Daniel Windschall, St. Josef-Stift Sendenhorst, Abt. Kinder- und Jugendrheumatologie, Sendenhorst; Asklepios Klinik Weißenfels, Weißenfels; Carl Thiem-Klinikum, Cottbus; Helios Kliniken Schwerin, Zentrum für Kinder- und Jugendmedizin, Schwerin; Klinikum Chemnitz, Klinik für Kinder- und Jugendmedizin, Chemnitz; Klinikum Mutterhaus der Borromäerinnen, Trier; Marien Hospital Witten, Witten; Städtisches Klinikum Brandenburg, Klinik für Kinder- und Jugendmedizin, Brandenburg an der Havel; Universitätsklinikum Giessen-Marburg, Marburg.
